# A Rights-based Approach to Information in Humanitarian Assistance

**DOI:** 10.1371/currents.dis.dd709e442c659e97e2583e0a9986b668

**Published:** 2017-09-20

**Authors:** Daniel P. Scarnecchia, Nathaniel A. Raymond, Faine Greenwood, Caitlin Howarth, Danielle N. Poole

**Affiliations:** Harvard Humanitarian Initiative Signal Program on Human Security and Technology, Harvard T.H. Chan School of Public Health, Cambridge, MA, USA; Harvard Humanitarian Initiative Signal Program on Human Security and Technology, Harvard T.H. Chan School of Public Health, Cambridge, MA, USA; Harvard Humanitarian Initiative Signal Program on Human Security and Technology, Harvard T.H. Chan School of Public Health, Cambridge, MA, USA; Harvard Humanitarian Initiative Signal Program on Human Security and Technology, Harvard T.H. Chan School of Public Health, Cambridge, MA, USA; Harvard Humanitarian Initiative Signal Program on Human Security and Technology, Harvard T.H. Chan School of Public Health, Cambridge, MA, USA

## Abstract

Crisis-affected populations and humanitarian aid providers are both becoming increasingly reliant on information and communications technology (ICTs) for finding and provisioning aid. This is exposing critical, unaddressed gaps in the legal and ethical frameworks that traditionally defined and governed the professional conduct of humanitarian action. The most acute of these gaps is a lack of clarity about what human rights people have regarding information in disaster, and the corresponding obligations incumbent upon governments and aid providers.  This need is lent urgency by emerging evidence demonstrating that the use of these technologies in crisis response may be, in some cases, causing harm to the very populations they intend to serve.  Preventing and mitigating these harms, while also working to responsibly ensure access to the benefits of information during crises, requires a rights-based framework to guide humanitarian operations. In this brief report, we provide a commentary that accompanies our report, the Signal Code: A Human Rights Approach to Information During Crisis, where we have identified five rights pertaining to the use of information and data during crisis which are grounded in current international human rights and customary law. It is our belief that the continued relevance of the humanitarian project, as it grows increasingly dependent on the use of data and ICTs, urgently requires a discussion of these rights and corresponding obligations.

## Humanitarian use of digital data: the coming crisis

Humanitarian assistance is increasingly digital data-driven and dependent on information communication technologies (ICTs). Humanitarian actors increasingly use ICTs as a core component of their work, including satellite and drone imagery analysis and mobile surveys. Meanwhile, crisis-affected populations increasingly rely on mobile technology to maintain contact with loved ones and diasporas, locate and access aid, transfer money, and identify migration routes.^1^

These developments, represent a pivot point in the history of the humanitarian project that requires new doctrine and norms. In this brief report, we provide a commentary that accompanies our report, the *Signal Code: A Human Rights Approach to Information During Crisis,*^2^ and seek to identify and explain the role that a rights-based approach to data and ICTs may have in retrofitting current humanitarian practice to meet the challenges it now faces.

Humanitarians have faced such consequential moments before, transforming the field both for better and for worse. The failures of the humanitarian response during the 1994 Great Lakes refugee crisis began an internal process which lead to the creation and adoption of the Humanitarian Charter and Sphere.^3^ These standards, which are rooted in a rights-based approach,^4^ drove the development of professional ethics and minimum technical standards which today shape humanitarian capacity and competence around five recognized forms of critical assistance: food, sanitation, shelter, protection, and healthcare.

The proliferation of ICTs among affected populations and humanitarian actors alike exposes critical, unaddressed gaps in the legal and ethical frameworks that have traditionally defined and governed humanitarians’ professional conduct.^5^ These gaps are an open secret, as is the lack of professionalization around data protection and ICT use. Increasingly, they are a disaster waiting to happen.^6^

The current largely unregulated use of digital data and ICTs by humanitarians may be changing how disasters unfold by causing secondary “big data disasters.” These types of secondary crises events, related to how data is created in process, can include “data deluge”, when responders are unable to process large amounts of data, and can also include what Raymond and al Achkar call “data damage.”^7^ This phenomena occurs when the irresponsible use of data harms crisis-affected populations and undermines the trust between responders and the communities they intend to serve. While these problems are complex, they fundamentally stem from the humanitarian community's failure thus far to comprehensively address the unique ethical and operational challenges caused by the data revolution.

There is emerging evidence that these operations may, in some cases, harm the very populations they intend to assist, violating human rights in the process. In one example, Amnesty International USA’s 2007 ‘Eyes on Darfur’ satellite program monitored select villages under threat of attack and atrocity. The Government of Sudan was notified of the project and warned that evidence of alleged atrocities would be made public. While the stated goal of the project was to reduce violence in the monitored villages, a 2016 study showed that violent acts against those villages later increased because the Sudanese government deliberately targeted these villages after the program concluded.^8^

In another example, humanitarian aid organizations and private sector partners used Call Detail Records (CDRs) to attempt contact tracing using aggregate mobile phone data during the 2014-2015 outbreak of Ebola in Liberia. In his 2016 paper *Ebola: A Big Data Disaster*, Sean Martin McDonald notes that this use of personal data may constitute non-consensual human subject experimentation. In addition to potentially violating international human subjects protections, little to no clinical advantage in tracking Ebola’s spread was realized as a result of this experimental use of CDR data.^9^

## Addressing the rights gap: information as a humanitarian need

The urgent need for an accepted rights-based framework and approach to these activities is becoming painfully clear. The most acute gap in current humanitarian doctrine is a lack of clarity about what human rights people have relating to information in disaster, and what obligations humanitarian actors, governments, and the private sector have for realizing these rights.^10^ In response, we have have recently published the *Signal Code: A Human Rights Approach to Information in Crises*, which identifies five human rights pertaining specifically to information and data in the context of natural and manmade disasters.^2^

These rights are provided for in current human rights law and custom, most notably Article 3 of the UDHR, which provides all people with the right to life, liberty, and security of person.^11^ They are already encoded into international humanitarian and human rights law, drawing on the same sources as the Humanitarian Charter and the Sphere Handbook. The rights are interdependent and interrelated: none can be realized individually without the realization of the others.^12^

The rights identified by the Signal Code are:

1. The right to access, generate, communicate, and benefit from information during crisis;
2. The right to protection from threats and harms resulting from the use of information and communications technologies and data during crisis;
3. The right to data privacy and security;
4. The right to data agency; and
5. The right to redress and rectification.

Preventing and mitigating the harms discussed above, while also working to responsibly ensure access to information during crises, begins with recognition that these rights exist and that they should guide how humanitarian information activities are conducted. Only from this initial articulation of rights, we believe, can a humanitarian practitioner’s obligations be appropriately codified and minimal technical standards for the use of data and ICTs later developed.

## The Future of Humanitarianism

The agreement of new international human rights standards and normative frameworks may seem like a naïve proposition in an era when international humanitarian law is being violated with apparent impunity in Syria, South Sudan, and elsewhere. However, as all aspects of humanitarian aid increasingly depend on the use of digital data and ICTs in some way, the future relevance of the humanitarian project as a whole will hinge on whether and how these questions are addressed. The human rights related to information during crises identified by the Signal Code are not, by themselves, a solution to these challenges, but it is our belief that they represent an essential step forward.

## Corresponding Author

Daniel P. Scarnecchia

dscarnecchia@hsph.harvard.edu

## Funding

The terms of the university's agreement with the funder are that they remain anonymous. The funder had no involvement in the writing of the manuscript or the decision to submit for publication.

## Competing Interests

The authors declare no conflicts of interest.

## Data Availability

There is no underlying data used in this paper.
